# Complication of solar branding: Report of a case and the review of the literature

**DOI:** 10.1002/ccr3.1847

**Published:** 2018-11-20

**Authors:** Mohammed Asif, Luis Quiroga, Anthony Sabo, Julie Caffrey

**Affiliations:** ^1^ Johns Hopkins University School of Medicine Johns Hopkins Burn Center Baltimore Maryland

**Keywords:** electrosurgery, skin grafting, wound healing

## Abstract

Branding is a type of scarification, a body modification that permanently transforms the skin by causing a visible scar. For centuries, it has been used on the skin of animals and slaves as well as criminals to convey ownership and also as a proof of guilt. More recently (in the 20th and 21st centuries), branding has become a symbol of personal identity, rites of passage, spiritual beliefs, and body decoration in some particular microcultures. Different means have been classically used to perform the desired branding designs including electrocautery, laser, chemicals, freezing, or a heated metal stencil. Solar branding is a new concept that involves using a focusing lens and a light source, usually the sunlight, to induce thermal injury. It is an emerging technique to perform body modifications and tattooing. As with other types of branding, solar branding also has its complications which may require surgical excision and grafting. We present a case of an acute skin infection following solar branding body modification, which to our knowledge is the first case reported in the literature.

## 1 INTRODUCTION

The concept of body modification by using the branding technique to express oneself is an old practice which has gained significant popularity among the younger generation in recent years.^1^, ^2^ Branding is performed with sterile equipment to inflict a third‐degree burn to the desired area of the body which eventually forms a permanent scar giving a unique and natural body art look.^2^ Several different branding techniques have been used to alter the human body which includes electrocautery, laser, chemicals, freezing, heated metal stencil, and, most recently, solar branding.^1^, ^2^, ^3^ Solar branding is a new concept that involves using a focusing lens and a light source to induce thermal injury. It is an emerging technique to perform body modifications and tattooing. As with any burn injury, there is a risk of hypertrophic or keloid scar, infection, full thickness skin loss, and delayed healing.^4^ We present a case of a skin infection following solar branding body modification, which to our knowledge is the first case reported in the literature.

## 2 CASE REPORT

A 32‐year‐old white woman, who was undergoing transgender body modifications and no previous medical history, presented to our emergency department (ED) with a chief complaint of right forearm pain, associated with redness and blistering. The patient was a tattoo fanatic who underwent solar branding on her right forearm approximately 2 weeks before presentation. The patient first noticed the blistering of the branded area 3 days after her body modification. Over the next few days, the entire branded area became very warm, swollen, painful to touch, and developed cellulitis with weeping blisters. Given her dire situation, the patient finally sought medical care in the local urgent care center. The patient was diagnosed with a superficial infection of her right forearm full thickness burn and prescribed oral clindamycin. The patient continued to develop painful blisters for the next week and did not notice any improvements in her cellulitis despite the antibiotic treatment, prompting her to come to our ED for a second medical opinion.

**Figure 1:**
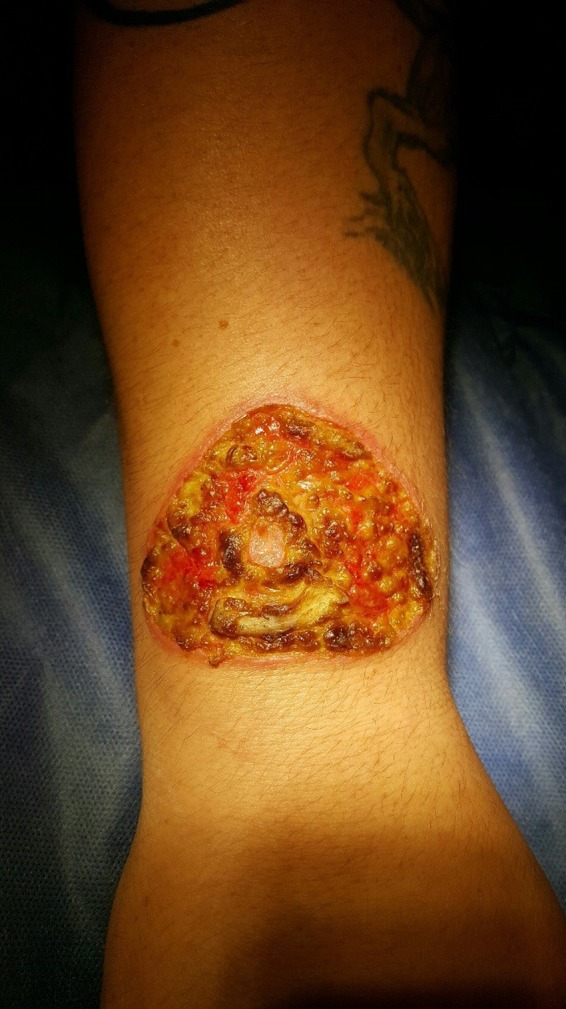
Painful blisters, redness, and swelling of the right forearm and hand 2 wk post–solar branding procedure

During her physical examination, the patient was noted to have painful blisters, redness, and swelling of the right forearm and hand (Figure 1). She did have a full range of motion of the hand; however, there was mild pain noted on the flexion of the hand. There were no signs of sepsis, and her vital signs and her laboratory panel which included white blood cell count were all within normal limits. The rest of the physical examination was also unremarkable. The patient was admitted to the Burn Surgery Service for intravenous (IV) antibiotics and possible operative interventions.

The patient was started on IV clindamycin 600 mg every 8 hours, and once adequate pain control was achieved, a bedside debridement was performed in the Burn Unit. The following day, the patient was taken to the operating room for tangential excision of her wound and split‐thickness skin autograft (STSG) placement onto her right forearm. The left anterolateral thigh was chosen as the donor site. The grafted site was dressed with xeroform, followed by a layer of bacitracin ointment and then wrapped with Kerlex and Coban dressings. A right forearm elbow splint was fitted and placed by the occupational therapy service for further graft protection. Postoperatively, the patient was transferred back to the floors without complications, and antibiotics were stopped. The right forearm dressing was taken down on postoperative day 4 to evaluate the STSG. The entire graft was viable and taken. Subsequently, the graft staples were removed on postoperative day 5.

**Figure 2:**
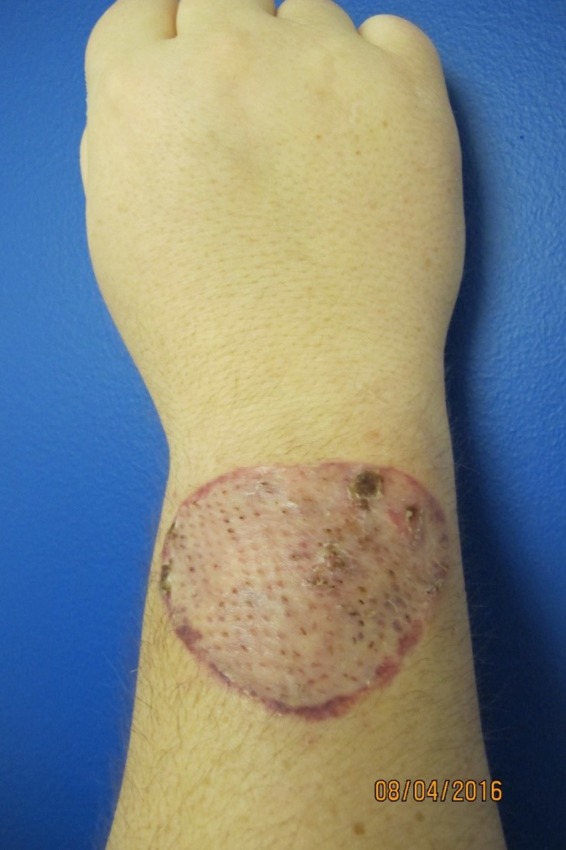
Hundred per cent split‐thickness skin graft take, 2 wk post–excisional debridement and autografting

The patient recovered exceptionally well and was discharged home a few days later. Two weeks later, the patient returned to the burn clinic for a follow‐up appointment. The skin graft and the donor site healed remarkably well (Figure 2). The patient reported no pain and had a full range of motion of the hand and wrist. The patient was discharged from the burn service and advised to use over‐the‐counter moisturizer or lotion as needed.

## 3 DISCUSSION

Different types of branding have gained significant popularity in particular groups of our society and fundamentally symbolize a counterculture that tries to differentiate from the mainstream. Branding, like other body modifications, has been attached to personal and sometimes spiritual beliefs. It allows people to not only self‐express their identity and make themselves visible in our large cities (communities) but also represents the ability to communicate with others through the particular meanings of body art. Branding of the skin represents a sign built on specific values, with equal aesthetic and symbolic components. Although branding is an old phenomenon, the recent innovation of solar branding has brought new ideas for body modifications. Like traditional branding, it requires burning of the skin by using sunlight. Because many factors are involved in the process of solar branding, miscalculation of the time and intensity of the light can produce easily unexpected and undesirable burn lesions and scars.^5^, ^6^, ^7^

For most people, the questions will be as follows: Is solar branding safe? and Is sterility a critical component to avoid infections? Historically, acute and chronic infections, delay scar healing, and keloid scars have been concerns for people undergoing branding via different techniques.

For those who undergo scarification by branding, directions for postoperative care are minimal including washing with soap and water and, leaving open to air and application of over‐the‐counter antimicrobial ointments. Wound management in these patients can be challenging as the injury itself tends to be deeper than what would be apparent on an initial examination.

Early identification of deep and infected burn wounds can be a challenge, and they are often disregarded by inexperienced observers as part of the natural healing process after this procedure. Systemic infections should be treated with antibiotics, and early treatment with debridement, irrigation, and application of silver sulfadiazine is appropriate. Tangential excision and autografting hasten healing, reduce disfigurement, and decrease costs to the healthcare system.

## 4 CONCLUSION

Early identification of burn wound complications after solar branding represents a great challenge for most medical health practitioners as it is not a common cause of burn. As this case illustrates, a significant delay in the final wound treatment could increase the chance of burn wound infection, conversion to a deeper wound, and the need for surgical excision and skin graft.

## CONFLICT OF INTEREST

None declared.

## AUTHOR CONTRIBUTION

MA, LQ, AS, JC: contributed to this article and approved its final version.
